# Enhanced Bioavailability and Dissolution of Atorvastatin Calcium from Floating Microcapsules using Minimum Additives

**DOI:** 10.3797/scipharm.1104-26

**Published:** 2011-11-05

**Authors:** Furquan Nazimuddin Khan, Mohamed Hassan G. Dehghan

**Affiliations:** Y. B. Chavan College of Pharmacy, Department of Pharmaceutics, Dr. Rafiq Zakaria Campus, Rauza Bagh, Aurangabad-431001, India

**Keywords:** Atorvastatin calcium, Bioavailability, Floating, Microcapsules, Pharmacokinetics

## Abstract

Atorvastatin calcium, a lipid-lowering drug, is much less bioavailable because of reduced solubility in acidic media. Multiple-unit floating microcapsules of Atorvastatin calcium (ATC) were developed to expand the gastric residence time of the drug, as ATC has maximum rate of absorption in the upper GI tract. Floating microcapsules were prepared by Emulsion-solvent evaporation technique through incorporation of dioctyl sodium sulphosuccinate (DSS) as a dissolution enhancer. The microcapsules were assessed for shape, size, drug entrapment efficiency, stability and *in-vitro* drug dissolution rate and were subjected to SEM, DSC and PXRD studies. The ATC-loaded floating microcapsules were spherical in shape and had the particle size of about 28.10 μm and drug-loading efficiency of about 96.55 %. The floating microspheres containing DSS had significantly higher drug dissolution rates than those without DSS. The best formulation, AT4, consisting of Ethyl cellulose, DSS and Poly Ox®, had a maximum drug dissolution rate of 97.86 %, as compared to Storvas 80 mg (Ranbaxy Ltd, as a reference) which had a rate of only 54% during a period of 12 h in acidic media. A pharmacokinetic study performed on albino rabbits illustrates that the bioavailability of AT4 floating microcapsules significantly increased to nearly 1.7 times that of Storvas 80 mg. The present study indicates that the use of multi-unit floating microcapsules for delivery of ATC can improve its bioavailability.

## Introduction

Atorvastatin calcium (ATC) is the treatment of choice in moderate to severe familial or non-familial hypercholesterolemia. ATC has a maximum rate of absorption in the upper GI tract, but because of poor solubility in this region, it is less bioavailable. Unpredictable and short gastric emptying time can result in incomplete drug release from the drug delivery system above the absorption region, which may be the stomach or upper component of the small intestine, leading to a reduced systemic availability of the administered dose. A previous study investigating improved solubility, stability and bioavailability of ATC includes a tablet formulation containing a complexing agent (cyclodextrins) and a surfactant (d-alpha tocopherol polyethylene glycol 1000 succinate) [1]. A self-micro emulsifying drug delivery system (SMEDDS) of ATC consisting of Labrafil, propylene glycol and Cremophor RH40 has been developed [2]. Recently, another self-emulsifying drug delivery system (SEDDS) of ATC in various vehicles such as Captex 355, Captex 355 EP/NF, Ethyl oleate, Capmul MCM, Capmul PG-8, Gelucire 44/14, Tween 80, Tween 20, and PEG 400 has been reported [3]. All these drug delivery systems have the disadvantage of complex manufacturing procedure and use of expensive additives with additional equipment and apparatus. The negative aspect of formerly reported solo unit systems such as tablets and capsules are the high inconsistency of their GI transfer moment because of their all-or-nothing emptying course of action. To overcome these limitations, multiple-unit floating microcapsules have been developed to form cost-effective stable ATC formulation with enhanced bioavailability. Multi-unit floating microcapsules will ensure a complete and constant drug release, particularly for drugs absorbed in the specific gastric area [4]. Such a dosage form can be circulated extensively throughout the gastrointestinal tract (GIT), providing a longer and more dependable release of the drug from the dosage form. Therefore, the focus of this research was to develop solubilized gastro-retentive multi-unit floating microcapsules of ATC to achieve better bioavailability.

## Materials and methods

ATC was obtained as a gift sample from Lupin India Ltd. Ethyl cellulose (Aqualon® N50), Hydroxy propyl methyl cellulose (Metolose® K4M) and Polyethylene Oxide (Poly Ox® N10) were provided as a gift sample by Colorcon Asia Pvt Ltd, Hydroxy propyl cellulose (Klucel G® 300) was supplied as a gift sample by Hercules Aqualon division. Light paraffin (LLP) and petroleum ether (40–60°) were purchased from Merck (India) and Dioctyl sodium sulphosuccinate (DSS granular) from Cytec Industries Inc.; all other chemicals were of analytical grade.

### Initial development of microcapsules

Floating microcapsules of ATC were prepared by emulsion solvent evaporation technique using different polymers such as EC, HPMC, HPC and Poly Ox® (Drug: polymer: DSS, 1:4:2) Table 1. Microcapsules with EC were prepared by dissolving ATC in 2ml methanol; EC and DSS were dissolved in acetone (8ml). The two solutions were mixed and dropped into a solution of light liquid paraffin 200 ml containing 0.25% Sorbitan monolaurate. The solution was stirred using a digitally controlled mechanical stirrer for 3 h at 800 RPM. After evaporation, microcapsules formed were collected by filtration and washed three times with petroleum ether and dried under vacuum at room temperature overnight. While the HPMC and HPC microcapsules are prepared by the same procedure as that of EC microcapsules, the only variation is the mixture of ethanol and dichloromethane (1:1) used for dissolving HPMC and HPC. All microcapsule formulations were prepared in triplicate. The prepared microcapsules were mixed with microcrystalline cellulose 10% and lubricated with 0.1% Magnesium stearate. Then, the final blend was compressed into tablets on 8 station rotary compression machine (Rimek, R&D model, “D” tooling), using suitable punches and dies.

### In-vitro floating ability

Microcapsules approximately 500 mg were introduced in dissolution apparatus consisting of 900 mL of 0.1 N HCL with 0.02% tween 80. The RPM (revolution per minute) was set at 100 and stirred for 12 h. The buoyant and the settled microcapsules were recovered, dried and weighed separately. The percent buoyant microcapsules were calculated as the ratio of quantity of microcapsules that were floating and the total quantity of the microcapsules [5].

### Physical state determination

The surface morphology of prepared microcapsules was determined by Jeol scanning microscope Japan (JSM 6015). Random SEM images of microcapsules were taken to find out shape, structural integrity and porosity.

### Determination of drug content and encapsulation Efficiency

Microcapsules equivalent to 80 mg of ATC was crushed and extracted with methanol and after appropriate dilution with 0.1N HCl, it was subjected to drug content analysis UV-visible spectrophotometer (V630, Jasco, Japan) at a wavelength of 246 nm. Drug concentration was determined with the help of the calibration curve. The range of drug concentration in the calibration curve was 5–100 μg/ml [6]. The entrapment efficiency was determined using the following formula:

$$\text{Entrapment efficiency}=\frac{\text{Calculated drug concentration}}{\text{Theoretical drug concentration}}\times 100$$

### Differential Scanning Calorimetry

Thermal characteristics of drug and drug excipients mixture were studied using a differential scanning calorimeter to ascertain that the drug is in pure form and there is no chemical interaction between the drug and the excipients during manufacture and storage. DSC measurements were performed on a Mettler-Toledo 821 instrument at a heating rate of 5°C/min, starting from 40 to 300°C.

### Assessment of Crystallinity

In order to determine the physical state of the drug before and after microcapsule preparations, the XRD patterns of the pure drug and the formulations were investigated using an X-pert pro and Pro-Anac diffractometer, Netherlands. The samples were irradiated with monochromatized CuKα radiation, and the scanning range (2θ) was from 2–50°. The voltage and current were set to 30kv and 30mA, respectively. X-ray patterns were analyzed using an X-pert data collector and X-pert data viewer V-1.0 software.

### In-vitro drug dissolution study

*In-vitro* drug dissolution rate determined by USP type II (USP XXIV) dissolution test apparatus. The study was conducted at 37 ± 0.5°C with agitation speed of 100 rpm in 0.1 N HCL (900 ml) as a dissolution medium. The samples were collected and filtered at predetermined time points, replaced with fresh media. Drug content in the samples were estimated by Jasco (PU2080 pump) HPLC system, with reverse phase Hi Qsil, 250mm × 0.45 ID, 5μm C_18_ column, and mobile phase comprising acetonitrile and phosphate buffer pH 3 in the 70:30 ratio, respectively. The wavelength was set at 246 nm at a flow rate of 1ml per minute [5].

### Stability studies

Stability studies were carried out according to International Conference on Harmonization (ICH) guidelines. The samples were stored at 40 ± 0.5°C/ 75 ± 5% RH (relative humidity) and 25 ± 0.5°C/ 60 ± 5% RH for three months. The samples were withdrawn and evaluated for drug content and physical change [7].

### Pharmacokinetics of ATC floating microcapsules

The protocol for the animal study in prescribed proforma B was approved by the IAEC approval no: CPCSEA/IAEC/P’ceutics-05/2010-11/27. Albino rabbits of both sexes weighing 2–3 kgs were fasted overnight and divided into two groups. Conventional tablets of 40mg × 2 (Storvas (40 mg, Mfd by: Ranbaxy Ltd) as reference was administered to group A. Because one 80 mg conventional tablet is too big to be administered to rabbits, 2 tablets of 40 mg strengths were administered consecutively one by one to ease of administration, and AT4 tablets equal in strength to the 80 mg floating microcapsules were given to group B. Blood samples were collected at 0.25, 0.5, 1, 2, 3, 4, 6, 8, 12 and 24 h. Blood samples (3ml) were withdrawn from the marginal ear vein. Whole blood withdrawn was centrifuged to separate serum. 1 ml was pipetted, deprotinated and diluted with acetonitrile to make 0.1μg/ml of drug solution. Estimation of ATC was performed by high-performance liquid chromatography (HPLC) analysis [6]. Extracted drug samples of plasma were assessed with a Jasco (PU2080 pump) HPLC system, with reverse phase Hi Qsil, 250mm × 0.45 ID, 5μm C_18_ column, and mobile phase comprising acetonitrile and phosphate buffer pH 3 in the 70:30 ratio, respectively. The wavelength was set at 246 nm at a flow rate of 1ml per minute. The calibration curve was plotted between the concentration ranges of 10 to 100 μg/ml that showed a correlation coefficient of 0.9992. The retention time of the ATC acid was 4.2 min.

## Results and discussion

### Initial development of microcapsules

Microcapsules were prepared by solvent evaporation method. ATC have very low solubility characteristics of acidic media, and to facilitate drug release, a water-soluble polymer such as Poly Ox® or HPMC or HPC was used along with a water insoluble polymer such as EC. The effect of incorporation of polymers such as Poly Ox®, HPMC, HPC, and EC on microcapsules characteristics such as size, shape, entrapment efficiency, yield and drug content was determined. The different formulations prepared are listed in Table 1. Microcapsules prepared by the EC alone were consistent, spherical and regular, whereas with HPC and HPMC alone or in combination with EC had irregular shape and were inconsistent. The irregular shape of microspheres consisting of HPMC or HPC may be because of a reduction in external volume as a result of the drying process. Quick water loss and heating promotes strains in the cellular structure of the materials, leading to a change in shape by means of shrinkage and formation of a concave surface [8]. Incorporation of Poly Ox® at 50 mg concentration did not affect the overall microcapsule characteristics such as shape and consistency as compared to HPMC and HPC. Based on these observations, EC and Poly Ox® polymers were selected for further studies.

A 3^2^ factorial design was used to design the batches of microcapsules for optimization studies. The two independent variables selected were amount of drug and amount of polymer, and their effect on drug dissolution rate was observed. All the floating microcapsule formulations were compressed into tablets and had good physical parameters such as hardness, friability, weight variation, disintegration etc. The prepared tablets were made to deliver microcapsules to the stomach, where the tablet disintegrates and releases the microcapsules in the stomach. As soon as these microcapsules are released in the stomach they float instantaneously in the acidic media. The physical parameters of formulations AT1to AT9 such as hardness and friability were satisfactory with % friability and disintegration time below 0.2% and 60 seconds, respectively, as shown in Table 3.

### In-vitro floating ability

The buoyancy percentage of the microcapsules observed was in the range of 74.33–91.16 ± 1.22% for formulation AT1 to AT9 at the end of 12 h. Tween 80 was incorporated in the medium for the wetting effect, to simulate the natural surface-active agents in the GIT. Microcapsules floated for an extended period of time on the surface of the medium without any noticeable gelation. The nature of the polymer influenced the floating behavior of the microcapsules. The results of % buoyancy for formulations AT1to AT9 are given in Table 2. The microcapsules containing higher polymer concentrations have high density and are less buoyant than those with lower polymer concentrations [9].

### Determination of drug content and encapsulation efficiency

Drug content of all the microcapsule formulations was above 80% as reported in Table 1. The highest drug content of 97.59 % was observed in EC microcapsules (M1) because of good encapsulation efficiency. The M2 and M3 formulations containing HPMC:EC and HPMC:HPC, respectively, resulted in low drug content which might be because of low encapsulation efficiency, as the microcapsules formed by these polymers are irregular and distracted. When EC was used in combination with HPMC (M4) and HPC (M5), the drug content was increased, but it was less than EC (M1) alone. Good entrapment efficiency of 93.22 % was recorded for M1. However, drug content of HPMC and HPC can be increased by varying HPMC:HPC ratio (1:4), but this resulted in a tacky and soft formulation.

The quantity of EC used on drug encapsulation efficiency revealed that, as the polymer quantity increased, encapsulation efficiency decreased (Batch no: AT3, AT6 and AT9). The emulsion-solvent evaporation technique using EC (AT4) resulted in microcapsules with good encapsulation efficiency of 96.55%.

### Physical state determination

**Fig. 1:**
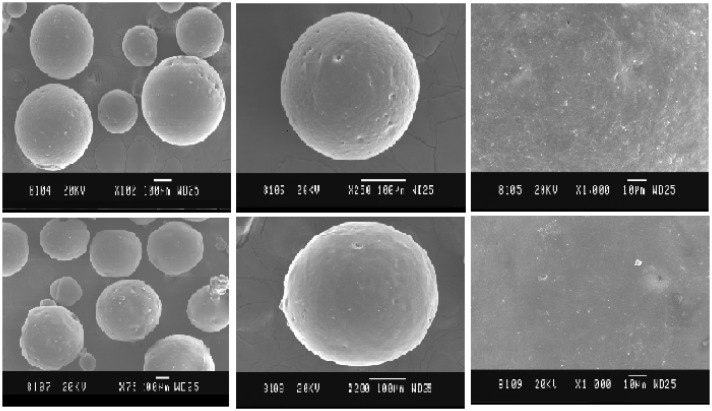
SEM of Blank EC Microcapsules (A, upper left); SEM of single Blank Microcapsule (B, upper middle); SEM of surface topography of blank EC Microcapsule (C, upper right); SEM of ATC loaded Microcapsules (D, bottom left); SEM of ATC loaded single Microcapsule (E, bottom middle); SEM of surface topography of ATC loaded Microcapsule (F, bottom right).

The diameter of all the batches except EC microcapsules was above 100 μm. As the concentration of EC increased, the microcapsule yield decreased. The results were similar when HPC and HPMC microcapsule were prepared. The particle size of HPMC microcapsules was 150 ± 35.22 μm and 171 ± 35.22 μm for HPC was observed at a polymer: drug ratio of 4:1, as the polymer concentration was decreased, more distracted particles were observed for both the polymers. The morphology of the factorial batches shows that the size of the microcapsules was controlled by EC concentration. The particle size increased with increasing EC concentration (AT9). The particle size of EC microcapsules was between 26.80 to 45.43 μm, as shown in Table 2. An SEM image shows that microcapsules obtained were spherical and no surface drug absorption on loaded microcapsules was observed. This demonstrates that the drug is equally distributed within the polymer coat, Figure 1. The mean particle size of the microcapsules increased considerably with increased polymer concentration, which may be because of high viscosity of the system at a greater polymer concentration leading to enhanced interfacial tension and reduced shearing efficiency [4]. The SEM images of single blank EC microcapsules showed surface porosity, which could be attributed to rapid solvent evaporation during preparation. However, this was absent in AT4 formulation because of a change in boiling point of acetone by the presence of ATC.

### Differential scanning calorimetry

**Fig. 2:**
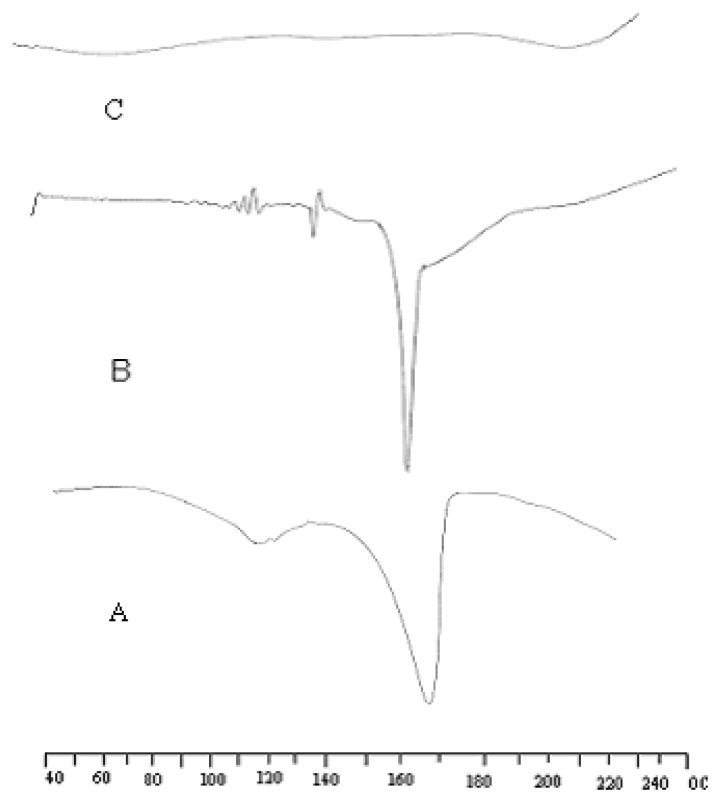
DSC thermogram of pure drug (A), AT4 microcapsules (B), Placebo without drug (C).

To determine the physical character of the drug before and after microcapsule formation, DSC analysis was performed for the pure drug, microcapsule formulation and placebo. Thermograms of the single component and final formulation are shown in Figure 2. DSC curve A of pure ATC showed an endotherm of water loss in the temperature range of 90–120 °C and a sharp endotherm at 164°C suggested the melting point of ATC. Curve B is the AT4 microcapsule formulation, which shows the melting transition at 168°C indicates that the drug is still in the crystalline form and the absence of endotherm of water loss in the temperature range of 90–120 °C in microcapsule formulation may be because of the conversion of trihydrate form to anhydrous form [10]. Slight shifting of the peak may be caused by the presence of other excipients. Curve C is the thermogram of placebo.

### Powder X-ray diffraction (pXRD)

**Fig. 3:**
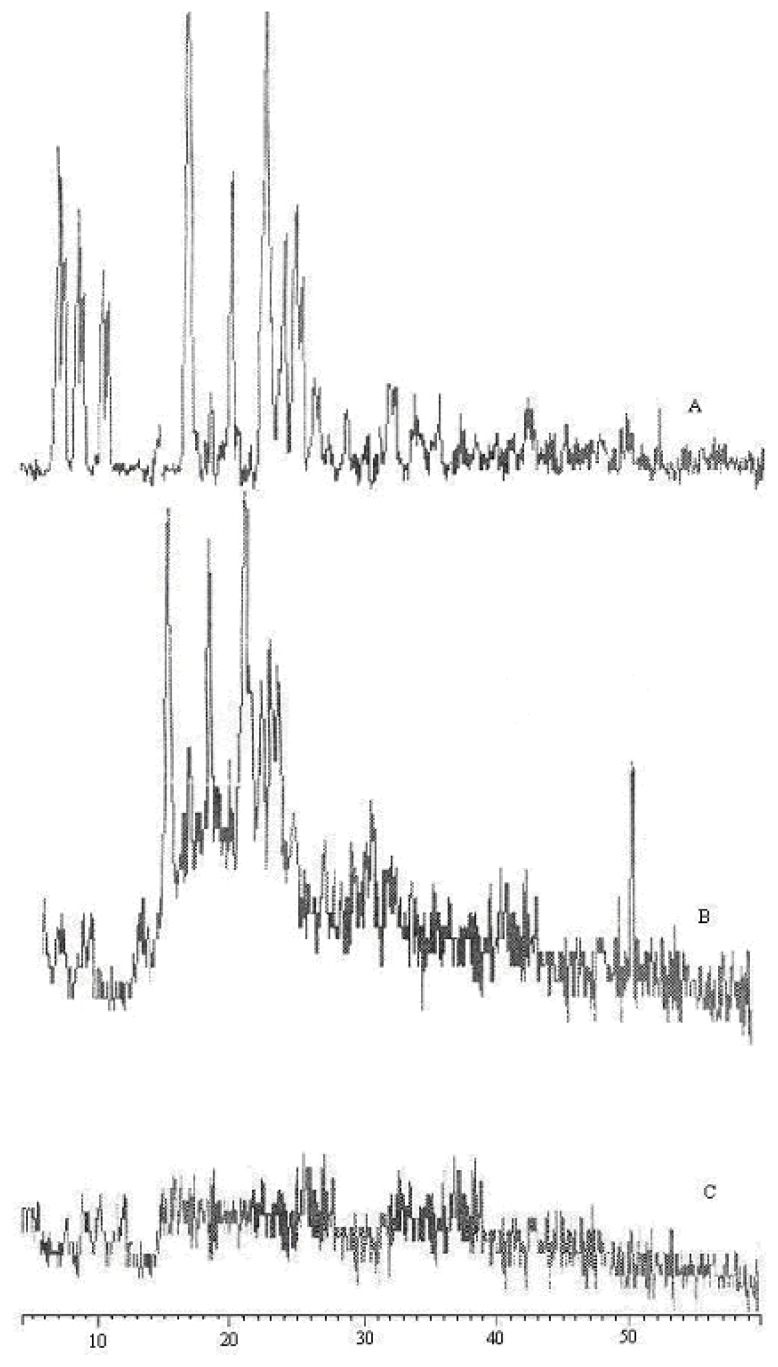
XRD diffractograms of pure drug (A), AT4 microcapsules (B), Placebo without drug (C).

The diffraction pattern of pure drug showed characteristic high-intensity diffraction peaks at 8.96, 9.35, 10.01, 10.37, 11.65, 12.00, 16.85, 19.26, 21.36, 22.50, 23.12, and 23.51, which indicates that the drug is present in the crystalline form that is also confirmed by DSC results, whereas AT4 microcapsule formulation showed reflections at 17.18, 18.66, 21.08, and 23.3. The pure drug exhibits reflections at 8.9, 9.3 and 10, these strong reflections of pure drug were masked in the microcapsule formulation and exhibits weak reflections at 8.9, 9.3 and 10 (two theta) 2θ. This decrease in intensity of reflection is attributed to the presence of other excipients in the formulation [11], Figure 3. It is clear that the reflections of the pure drug match satisfactorily with the reflections of the drug in the microcapsule formulation. Thus, it can be concluded that the polymorph of pure drug was the same as that of ATC polymorph incorporated in microcapsules, and no transformation took place during the manufacturing process and storage.

### Drug dissolution studies

**Fig. 4:**
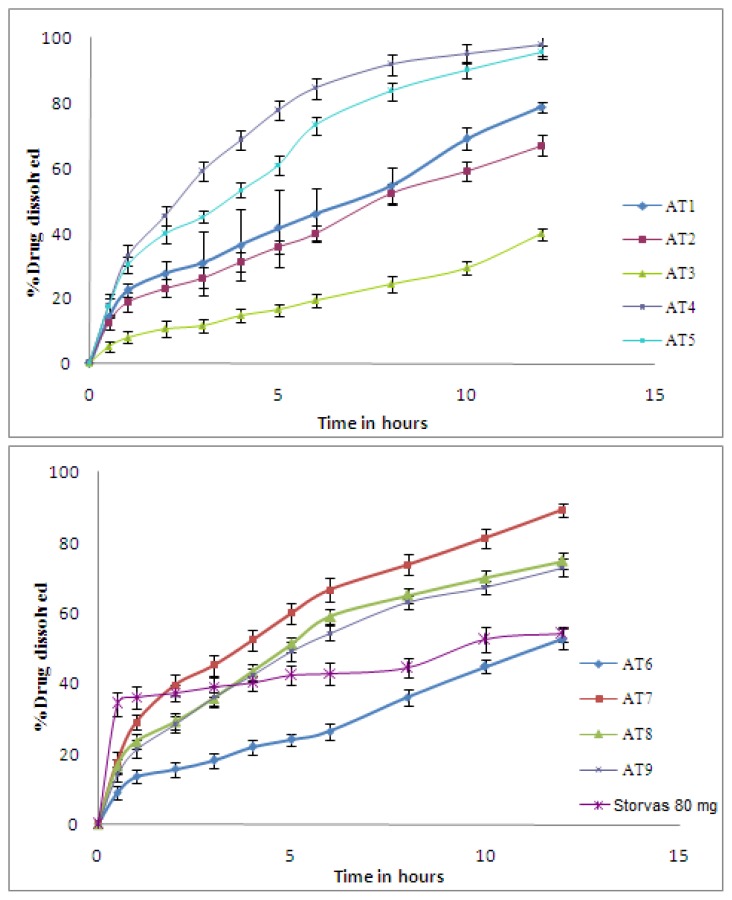
Dissolution profile of factorial batches from AT1 to AT9 and reference tablet Storvas 80mg in 0.1 N HCl.

The quantity of polymer affects the particle size as well as the dissolution of ATC from microcapsules. The polymer-drug ratio along with the amount of surfactant DSS may be the influential factor in the dissolution of ATC from the EC microcapsules. Different solubilizers such as DSS, Poly Oxyethylene Sorbitan Fatty Acid Esters (Tween 80), Sorbitan monolaurate (Span 20), polyethylene–propylene glycol copolymer (Polaxomer) etc., were used, and their effect on physicochemical properties of microcapsules was determined. Among all the solubilizers used, only DSS was able to enhance the dissolution of ATC in 0.1 N HCL with 80% of dissolution in 8 h, as compared to the other polymer mixtures and pure drug as shown in Table 4. DSS also helps in maintaining the integrity of the microcapsules during the development process and dissolution study. DSS is widely used as an anionic surfactant in pharmaceutical formulations to assist in wetting and dissolution, [12, 13]. Above 50 mg and 100 mg concentration of Poly Ox® and Poloxamer, the matrix of the microcapsules began to dissolve within 2–3 h of dissolution. Beyond certain concentrations of other polymers used in the dissolution study, it hampered the microcapsule formation in terms of shape and consistency. The drug dissolution percentage of Storvas 80 mg tablet was only 55% in 12 h of dissolution in acidic media as compared to the rate of ATC floating microspheres, which was 97.86%, 95.69% and 89.31% for AT4, AT5, and AT7, respectively. The drug dissolution significantly decreased with an increase in the amount of polymer in the microcapsules, and the higher density of the polymer matrix because of more concentrations gives rise to an increased diffusional pathlength. This may lead to decrease in overall drug release from the polymer matrix. Moreover, smaller microspheres are formed at a lower polymer concentration and have a larger surface area exposed to dissolution medium, giving rise to quicker drug release, Figure 4.

### Stability studies

Stability studies on optimized batch AT4 of EC microcapsules determined at 40 ± 0.5°C/ 75 ± 5% RH and 25 ± 0.5°C/ 60 ± 5% RH for a period of 3 months showed no physical change and drug content determined corresponded to the earlier initial results obtained before storing of microcapsules, as shown in Table 3. The anionic surfactant DSS provides the necessary stability to the formulation by maintaining the sufficient alkaline conditions during storage and dissolution studies. Therefore, it may be suggested that no chemical interaction between excipients and ATC took place.

### Pharmacokinetics of ATC floating microcapsules

**Fig. 5:**
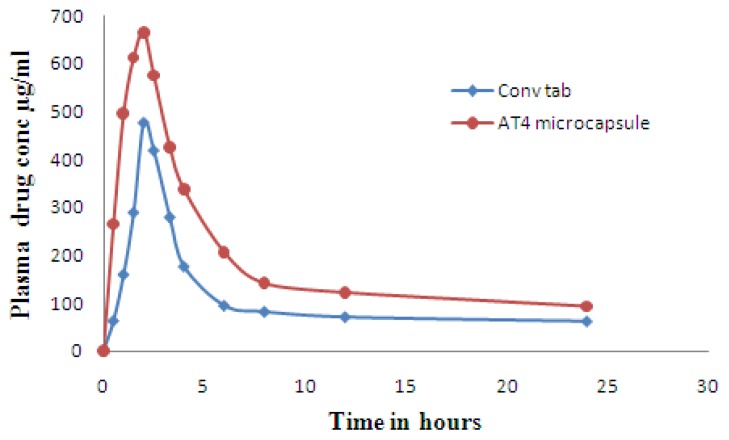
Plasma concentration time profiles of conventional tablet and AT4 microcapsule formulation.

The plasma concentration versus time profile of AT4 floating microcapsules and the conventional tablets is shown in Figure 5. Microcapsule formulation is well absorbed as there is a complete dissolution in acidic media owing to greater surface area for dissolution, without degradation of the drug when compared to conventional tablets, as Cmax of microcapsules is higher than that of conventional tablets (665 ng/ml for microcapsules and 478 ng/ml for conventional tablets). The pharmacokinetic parameters such as AUC and Cmax show significant differences between the two formulations. There were no significant differences between the two formulations for t_1/2_ and tmax. The C max and AUC_0→24h_ of the floating microcapsules were significantly higher than those of the conventional tablet. The relative bioavailability of AT4 formulation was about 1.7 fold compared with the conventional tablet, Table 5. This may be because of complete solubilization and better absorption of ATC. Therefore, ATC floating microcapsules can increase the oral bioavailability of ATC.

## Conclusion

The floating microcapsules of ATC-containing ethyl cellulose, Poly Ox®, docusate sodium, shows signs of good results with respect to incorporation efficiency, percentage yield, solubility and sustained drug dissolution rates. The AT4 formulation is capable of complete drug dissolution within 12 h. The developed floating microcapsules have the potential to enhance the oral bioavailability of ATC. After oral administration of ATC to rabbits, the bioavailability of ATC significantly increased by nearly 1.7 times compared with that of the conventional tablets. The present study shows that multi-unit floating microcapsules of AT4 are the best formulation for improved systemic availability of ATC.

## Figures and Tables

**Tab. 1:** Characteristics of the initial development of microcapsules.

Batch Nr.	Polymer:Drug	Yield	Average Diameter (μm)	Incorporation Efficiency (%)	Drug Content
M1	EC/ATC (4:1)	82	45 ± 12.45	93.22	97.59
M2	HPMC/ATC (4:1)	57	150 ± 35.22	87.01	91.81
M3	HPC/ATC (4:1)	60	171 ± 37.89	89.89	90.28
M4	EC: HPMC/ATC (4:4:1)	65	120 ± 28.56	92.12	95.64
M5	EC: HPC/ATC (4:4:1)	59	129 ± 31.65	91.38	95.12
M6	HPMC: HPC/ATC (4:4:1)	50	252 ± 26.44	82.65	89.33
M7	EC: Poly Ox® (4:0.5)	81	44 ± 39.45	94.04	96.98

**Tab. 2:** Factorial design batches with variables and their level.

Batch Code	X_1_	X_2_	Yield (%)	Particle size (μm)	Entrapment efficiency (%)	Buoyancy (%)
AT1	−1	−1	76.02±2.11	26.80±7.27	93.94±2.57	90.67±1.55
AT2	−1	0	71.72±2.32	28.51±5.30	95.67±3.61	85.00±1.26
AT3	−1	+1	98.50±1.84	32.39±9.63	84.51±1.07	76.33±1.47
AT4	0	−1	74.17±1.38	28.10±6.58	96.55±4.05	91.16±1.22
AT5	0	0	71.25±2.35	30.03±8.43	71.05±3.84	87.34±1.42
AT6	0	+1	95.64±1.89	36.29±5.39	86.99±2.01	78.67±1.39
AT7	+1	−1	73.32±1.71	29.61±5.41	79.33±1.85	84.54±1.77
AT8	+1	0	80.82±2.12	32.25±6.09	81.08±2.45	80.88±1.51
AT9	+1	+1	93.00±2.19	41.43±4.11	87.95±2.11	79.15±1.96

Amount of Drug X_1_ = +1 (150mg), 0 (100mg), −1 (50mg); Amount of Polymer X_2_ = +1 (450mg), 0 (400mg), −1 (350mg);

* Amount of DSS = 200 mg and Poly Ox® =50 mg were kept constant in all the batches; Drug content analyzed after 3 months of storage.

**Tab. 3:** Physical parameters and assay of tablets containing microcapsules of factorial design batches.

Batch Code	Hardness (kg/cm^2^)	Thickness (mm)	Friability (%)	Disintegration Time (Sec)	Drug content Initial (%)	Drug content After 3 M (%)
AT1	3.5±0.47	2.5±0.05	0.10±0.07	43.2±0.33	96.28±1.15	96.01±0.50
AT2	3.4±0.83	2.6±0.08	0.15±0.05	31.2±0.42	98.51±0.53	98.26±0.83
AT3	3.5±0.84	2.4±0.05	0.13±0.02	52.2±0.38	90.89±0.81	90.63±0.64
AT4	3.5±0.50	2.5±0.04	0.11±0.05	35.2±0.29	98.97±0.54	98.86±0.87
AT5	3.5±0.65	2.6±0.01	0.12±0.05	59.2±0.24	81.08±0.76	80.95±1.10
AT6	3.4±0.43	2.6±0.02	0.17±0.04	45.2±0.41	89.43±1.22	89.22±1.05
AT7	3.4±0.41	2.5±0.01	0.12±0.06	30.2±0.32	87.54±1.09	87.41±0.93
AT8	3.6±0.87	2.4±0.06	0.14±0.03	40.2±0.29	89.88±0.85	89.71±0.81
AT9	3.5±0.86	2.4±0.07	0.15±0.07	50.2±0.37	92.13±0.94	92.05±0.64

**Tab. 4:** *In-vitro* drug dissolution studies

EC:ATC	DSS	Citric Acid	Span 80	PEG 4000	Poloxamer 407	Poly Ox® N10	Tween 80	Drug dissolution at 8th hour (%)
400:100	200	–	–	–	–	–	–	80 ± 0.64
400:100	–	50	–	–	–	–	–	5 ± 0.57
400:100	–	–	100	–	–	–	–	7 ± 0.31
400:100	–	–	–	100	–	–	–	26 ± 0.43
400:100	–	–	–	–	100	–	–	52 ± 0.48
400:100	–	–	–	–	–	50	–	46 ± 0.52
400:100	–	–	–	–	–	–	50	35 ± 0.59
pure drug	–	–	–	–	–	–	–	10 ± 0.39

n = 3; Mean ± SD.

**Tab. 5:** In-vivo pharmacokinetic parameters of ATC and relative bioavailability of AT4 microcapsules and conventional tablets.

Formulation	Tmax (h)	Cmax (ng/ml)	AUC_0–24h_ (ng.h/ml)	Fr (%)
Conventional Tablet	2.00 ± 0.48	478 ± 24.22	2557.51 ± 319.57	100.00
EC microcapsule (AT4)	1.98 ± 0.25	665 ± 33.16	4490.22 ± 431.88	175.60

n = 6; Data are means ± SD.
